# Dynamic assessment of the risk of airborne viral infection

**DOI:** 10.1111/ina.12862

**Published:** 2021-07-02

**Authors:** Alessandro Cammarata, Giuliano Cammarata

**Affiliations:** ^1^ Dipartimento di Ingegneria Civile e Architettura Univerità degli Studi di Catania Catania Italy; ^2^ Dipartimento di Ingegneria Elettrica, Elettronica e Informatica Univerità degli Studi di Catania Catania Italy

**Keywords:** aerosol transmission, Coronavirus, pandemic, probability of contagion, SARS‐CoV‐2, virus airborne transmission

## Abstract

This paper applies the Rudnick and Milton method through the dynamic evaluation of the probability of airborne contagion, redefining all parameters and variables in discretized form. To adapt the calculation of the risk of contagion to real needs, scenarios are used to define the presence of people, infected subjects, the hourly production of the quanta of infection, and the calculation of the concentration of CO_2_ produced by exhalation in the air. Three case studies are discussed: a school, an office, a commercial activity. Complex scenarios include environmental sanitization, a variable number of people, and the possibility of simulating work shifts. The dynamic evaluation of the quanta of infection is also estimated, not foreseen by the Rudnick and Milton model, and involves updating the average values of the equivalent fraction of the indoor air with an improvement in the accuracy of the calculation due to the reduction of improper peaks of the stationary variables.

**Practical implications**

- The proposed method allows for better passive protection of infections by alternating work periods with full personnel and short rest periods.
- The proposed method with sensitivity analysis allows to determine the spread of individual probabilities due to the variability of the parameters.
- The method makes it possible to determine the best operating conditions for intervening on HVAC systems or for the insertion of CMV units both in individual rooms and in larger work areas.
- The use of a method based on the number of equivalent air‐changes allows to reduce energy and plant costs by meeting the requests of the major plant manufacturers.

## 1 INTRODUCTION

W.F. Wells was the first to hypothesize the mechanism of viral transmission by air through the emission from the mouth and nose of tiny organic droplets coming from the esophagus.^1^ These droplets are emitted by various mechanisms such as breathing, speaking, coughing, and sneezing. Droplet emission rates vary greatly from 2 to 4 (m/s) for breathing to 60–80 (m/s) and more for sneezing. Besides, the distances traveled can vary from a few meters to ten meters for coughing. The droplets, with dimensions of 10 (μm), evaporate^1^ in a few seconds forming the droplets nuclei with dimensions of a few μm, very light and such as to form an aerosol that can remain suspended in the air for a few hours. An update on droplet evaporation rate was done by Xie et al in 2007.^2^ The infection caused by the droplets emitted from the mouth and/or nose is direct, affecting the respiratory tract of the affected subjects and penetrating inside the bronchi and lungs (apart from other forms of contagion of other internal organs), Beggs et al., 2004.^3^ According to these recent studies,^4^ the particles emitted by expiratory activity have diameters ranging from 0.8 to 5.5 μm. Studies on this mechanism have been carried out.^5^, ^6^ The lighter particles, up to 5 (μm), affect the deeper regions of the respiratory tract up to the deep alveolar region, with the possibility of activating the contagion, while particles with a diameter >5 (μm) are trapped in the upper area of the respiratory track with lower probability of contagion.^7^ All this suggests that the probability of contagion associated with particles with a diameter greater than 5 (μm) must be carefully evaluated. This last scarce possibility of contagion led virologists, until the beginning of July 2020, to consider airborne viral infection to be negligible. The importance of transmission of the infection by air has been highlighted^8^ for the flu but similar results also apply to the SARS‐CoV‐2 virus. They also emphasize how good ventilation can reduce the epidemic, as can the vaccination of about half the people present. The close contagion is said to be of short distance^9^ and is significant, on average, within 2 (m) from the infected subject. Several intervention measures for the close‐range contagion are available, e.g.

- use of individual protection masks;
- interpersonal distance from 1 to 2 m, depending on the case;
- handwashing with disinfectants to avoid the indirect contagion of droplets that fall by gravity on horizontal surfaces.

There has been much discussion on the effects of aerosol, which remain suspended in the air inside closed environments.^10^ Recently, 239 scientists from around the world reiterated the possibility of infection from SARS‐CoV‐2 via aerosols, and the World Health Organization confirmed this hypothesis in July 2020.^11^ The contagion through aerosols acts at beyond the two meters typical of direct contagion, due to the circulation induced by the air inside closed environments.^12^ The aerosol is of no importance outdoors because it is immediately dispersed by air currents and diluted in a huge mass of air.

The infection risk due to airborne contagion can be evaluated by using the Wells‐Riley equation,^13^ with the concept of quanta emission and the dilution effects of viral loads due to ventilation in the rooms. Almost all models, proposed for calculating the probability of individual contagion, follow this approach, albeit with significant improvements. In this article, we want to present a calculation method that, using the basic idea of Rudnik and Milton^14^ of spreading the infection by re‐breathing the air inside the rooms by the susceptible people present. The air exhaled by the infected person contains viral loads which, in the re‐breathing phase of healthy subjects, can cause airborne contagion. Matthew J. Evans,^15^ carried out a risk analysis of airborne contagion arguing that normal return to work with the current COVID 19 epidemic is not possible until more than 1 in 1000 subjects is asymptomatic. Furthermore, he assumes that the assessment of the risk of airborne infection based only on the symptomatic infected is less than 1/1000 of the real one. The original method of Rudnick and Milton^14^ is here completed with the calculation of the variable hourly distribution of the quanta of infection, as done with the method of Gammaitoni‐Nucci in 1997,^16^ and enhanced using the discretized dynamic calculation and using profiles to adapt the calculation to real situations and needs.

## 2 EXISTING METHODS FOR CALCULATING THE PROBABILITY OF INDIVIDUAL CONTAGION BY AIR

From the considerable number of scientific publications on airborne viral infection, two methods of calculating the personal probability of infection emerge^17^:

1. Wells‐Riley method^13^;
2. Gammaitoni‐Nucci method.^16^

The more recent method proposed by Rudnick‐Milton^14^ has had some applications recorded in the literature,^18^ and some improvement proposa.^19^

All these three methods express the probability of individual contagion in the mathematical form of the Poisson curve for the statistical distribution of the contagion:

(1) $$p=1‐e^{‐\mu }$$

where *p* is the probability of contagion and μ is the Poisson factor. The factor μ includes some fundamental quantities, such as:

- the breathing activity of a single individual of ambient air, usually indicated with the letter *p*, in (m^3^/h);
- the number of initial infected, usually equal to 1, indicated with the letter *I*;
- the exposure time from entering the environment, indicated with the letter *t*, (h);
- the total flow rate of fresh air, *Q* (m^3^/h), calculated as the product of the number of hourly changes, *N_r_* (1/h), by the volume, *V* (m^3^), of the environment;
- the number of quanta of infection, (quanta/h), produced by the infected within the environment.

This last quantity has an epidemiological definition and contains two fundamental information: the number of viral loads and the term of infectivity, that is the probability that the viral load starts an infection.^4^, ^20^ In practice, for the quanta (*q*) the definition holds:

Definition 1 *Q* = number of quanta/unit time × term of infectivity.

Quanta are not easily calculated directly, also due to the great uncertainty of the RNA pairs, but are estimated epidemiologically from epidemic cases by replacing the calculated probability, *p*, by the number of infected.^21^ An alternative approach to the quanta method is the *dose*‐*response* method.^20^ The latter requires a sufficient amount of data to build a dose‐response relationship. The conceptual simplicity of the quanta is now criticized by epidemiologists because the infection depends not only on the viral load but also on the general health of the infected subject and the type of viral load absorption. The Wells‐Riley relationship,^13^ is as follows:

(3) $$p=1‐e^{‐\frac{\mathrm{Iqpt}}{N_{r}V}}$$

where all the quantities have been defined in the Nomenclature. For all the calculation methodologies the following considerations can be made, ceteris paribus:

1. The probability of individual contagion decreases as the volume of the environment increases;
2. The probability of individual contagion decreases as the number of hourly changes of fresh outside air increases;
3. The probability of individual contagion increases with increasing exposure time from the moment of entry into the environment.

The first observation depends on an architectural variable, the volume of the room *V*, on which we can hardly intervene. Consequently, in small rooms, it is good to stay for as little time as possible. The second observation depends on external air ventilation. This can be of two types: natural ventilation^22^ and forced ventilation.^23^

## 3 METHODOLOGY

### 3.1 Theoretical basis of the Rudnick and Milton method

The method of Rudnick and Milton,^14^ wanted to deepen and modify the calculation relationships proposed up to then exclusively based on the balance of the quanta of infection and the infected in the environments. The basic idea of the new method is that the virus in circulation is conveyed through the exhalation air of the infected which, subsequently, is re‐inhaled (*re*‐*breathing*) by susceptible subjects in the environment. The higher this breathing activity, the greater the possibility of transmitting the viral infection by air. Other research has been published in subsequent years,^18^, ^19^, ^24^ based on the re‐breathing mechanism and the use of CO_2_ as a marker. Due to its flexibility and ease of application, in the following description, only the Rudnick and Milton method will be recalled. Since the air exhaled by people inside the environment always contains, for the internal metabolism, a percentage of CO_2_, as well as a lower percentage of O_2_ compared to the inhaled air, it can be hypothesized to use the CO_2_ concentration to trace the breathing activity of the subjects inside the environment. The novelty of the method consists, in fact, in taking into account the people present in the environment and the amount of air breathed and re‐inhaled in repeated breathing cycles. The human body inhales a flow of air that depends on the age, the activity performed, the state of health of the subject, and the functioning of the pulmonary system. In all cases, in the exhalation phase, part of the oxygen present in the inspired air is replaced by CO_2_. Using appropriate sensors, it is possible to trace the global respiratory activity by tracing the CO_2_ emitted. Of course, the hypothesis is valid that within the environment there are no other sources of CO_2_ other than that of expiratory origin.

Other basic assumptions for the mathematical developments are as follows:

- limited room size to avoid vorticity effects and unevenness in air distribution. The simplifying hypothesis of perfect mixing is therefore valid;
- constant temperature and humidity in the calculation period;
- constant viral activity and survival time in the calculation period;
- calculation period limited to a fraction of a day (work shifts, lesson hours, commercial activity hours).

Unlike what indicated by Rudnick and Milton in their publication, instead of making a balance of CO_2_ fractions present in the ambient air of volume *V* and produced by the exhalation of the subjects in the same volume *V_e_*, a balance of the CO_2_ *concentrations* for the two volumes is provided. Therefore, in the hypothesis of well‐mixed ventilation (perfect mixing), it is possible to write a balance for the CO_2_ in the environment, i.e.

(3) $$C_{a}V_{e}=\left(C‐C_{0}\right)V$$

The advantage of this setting is to be able to correlate the concentrations, expressed in (ppm), to the ventilation air‐flow rate and therefore to the whole set: building‐HVAC system‐people. The dynamic method proposed in this article derives from this approach. Solving the previous equation for the ratio *V_e_*/*V*, we obtain:

(4) $$f=\frac{V_{e}}{V}=\frac{C‐C_{0}}{C_{a}}$$

where *f* is the *equivalent fraction of internal air* ^14^ which is exhaled and which is also the fraction of newly inspired air. Note the definition of *f* is valid for both steady‐state and transient regimes, evaluating the mean value instantly. For the total period of exposure, *t*, it is possible to calculate the average fraction of exhaled air, by integrating *f* in the same period considered. The equivalent fraction of indoor air, *f* can also be determined by the expression:

(5) $$f=\frac{V_{e}}{V}=\frac{{\overset{\cdot }{V}}_{e}}{\overset{\cdot }{V}}=\frac{N_{p}p}{\overset{\cdot }{V}}$$

being *V_e_* the volume of exhaled air and *p* the breathing rate. To calculate the difference $\Delta C=C‐C_{0}$, the steady‐state expression was taken into account:

(6) $$\Delta C\equiv C‐C_{0}=\frac{S_{g}}{N_{r}V}=\frac{p^{\prime }Np}{N_{r}V}$$

From the comparison between the previous expressions, we have the following expression:

(7) $$f=\frac{C‐C_{0}}{C_{a}}=\frac{p^{\prime }Np}{N_{r}VC_{a}}$$

with *C_a_* varying between 370,000 and 400,000 (ppm) in conditions of normal activity.The model follows the standard of all models for calculating the probability of infection, i.e: one (*I *= 1) initially infected person who emits all quanta of infection; *N_p_* susceptible infected persons, of whom only one is infected, who do not emit additional infection quanta. Re‐breathing relates to the transmission of quanta produced by the infected person to susceptible individuals who breathe in the infected air exhaled by the infected person. In these conditions the production of the quanta of infection, *N* (quanta/m^3^), is equal to the total concentration of the quanta in the air exhaled by the infected subjects, *q*/*p*, multiplied by the volumetric fraction of the air exhaled by the infected in the volume of the environment, $fI/N_{p}$, and therefore it is derived that:

(8) $$N=\frac{\mathrm{fIq}}{pN_{p}}$$

The average value over time *t* is:

(9) $$\bar{N}=\frac{\bar{f}Iq}{pN_{p}}$$

It is important to note that both expressions (8) and (9) have an apparent dependence on *N_p_*: by substituting the expression (7) into them *N_p_* disappears. Then, recalling the Poisson relation:

(10) $$p=1‐e^{\bar{\mu }}$$

replacing the calculated values in the exponent gives the probability of contagion:

(11) $$p=1‐e^{‐\frac{\bar{f}Iqt}{N_{p}}}$$

Even in the latter expression, the dependence of *N_p_* is only apparent. This equation remains valid in both stationary and transient conditions and may not depend on direct knowledge of the ventilation airflow, usually difficult to calculate in existing systems, but also on CO_2_ balances alone. It is sufficient to measure or calculate the CO_2_ produced inside and that of the external ventilation air to obtain the fraction *f* and, integrating the average value $f_{m}$ over time, calculating the probability of infection *p*.

### 3.2 Dynamic discretized version of the Rudnick and Milton method

**FIGURE 1:**
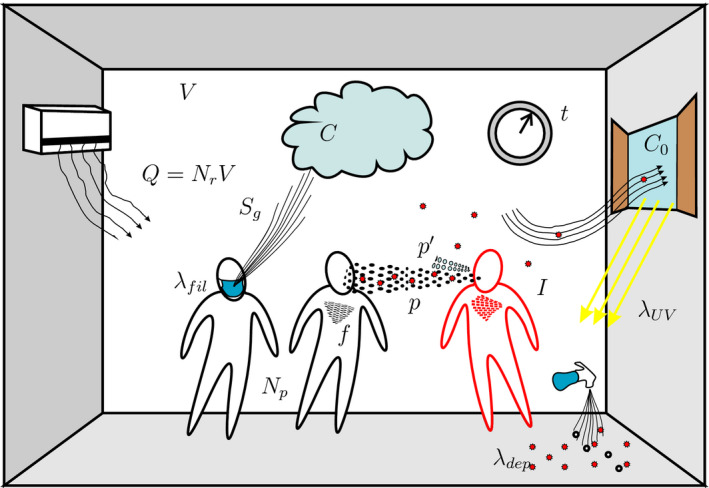
Sketch of airborne transmission in indoor

Some authors^19^ took up the idea of Rudnick and Milton proposing to directly calculate ΔC through a balance equation of CO_2_ in the environment and to calculate the equivalent fraction of indoor air, *f*, by Eq. (4). However, the CO_2_ balance equation is placed in a simplified form, and therefore it is preferred to set it more completely, as will be described in the following. Here, we want to propose another method of using the Eq. (11) for design and verification. ΔC can be directly calculated through a balance of the CO_2_ produced in the environment and, considering that the Eq. (11) is also valid in non‐stationary conditions, or in conditions that we define *discretized dynamics*, that is with calculation parameters and operating conditions that vary discretely over the calculation time. To be able to operate in this new model, some formal changes must be made to the Rudnick and Milton formulation. The quantities that appear in Eq. (11) can, in whole or in part, be discretized, that is, transformed into numerical sequences at a constant step according to a profile of use that indicates a desired, even non‐uniform, temporal variability. In these hypotheses the Eq. (11) becomes:

(12) $$p=1‐e^{‐\frac{f_{m}\bar{I}\bar{q}t}{{\bar{N}}_{p}}}$$

where the discretized parameters are gathered in vectors and expressed in boldface. In this way, it is possible to describe each calculation parameter and each variable through a sequence of numerical values independent of analytic relationships and freely modifiable as required. Notice that, in transient conditions, the probability *p* is evaluated considering averaged variables. The time is also discretized following the scenario. This makes it possible to obtain the desired time sequence, for example, to take into account double work shifts or to consider sanitizing the rooms during the work break to eliminate CO_2_ accumulations (a total change of ambient air) and of quanta of infection from the previous work‐shift (Figure 1).

**FIGURE 2:**
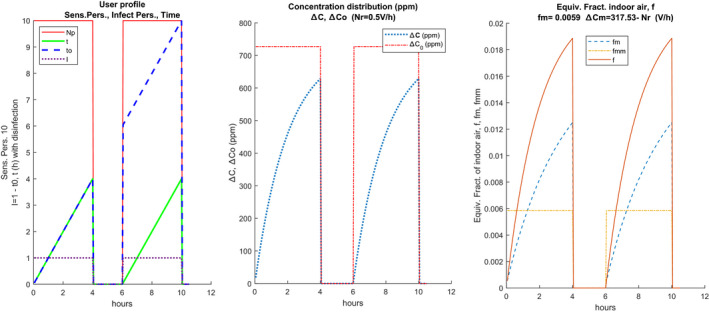
Example of scenario with intermediate disinfection

Figure 2 shows an example of a vectorized scenario in graphic form.

Starting from left, the following quantities are plotted:

- $N_{p}$ number of people present as time changes. In this case, the 17 people are present in two shifts of 4 h each with a break of 1.5 h;
- **I** the initial numbers of infected people present during the calculation, also in vectorized form. In the case in question, there is 1 infected (constant) at the beginning of each shift, having assumed that the room was sanitized during the break;
- $t_{0}$ is the progressive calculation time. In the case in question it does not appear during the intermediate break because there are no people or infected;
- ***t*** is the calculation time according to the scenario for each work shift. It always starts from zero at the beginning of each work shift, in the case of carrying out the sanitization of the room, otherwise, it would have the same trend as $t_{0}$. The calculation time, performs the discretized calculation under the choices made in the scenario. It applies to all the quantities calculated through Eq. (12): the probability of individual contagion, probability of global contagion, and hourly distribution of quanta.

Sanitization can be achieved by completely renewing the air inside the rooms (both naturally with the opening of the windows and mechanically with mechanically controlled ventilation or MCV system) sanitizing the surfaces where the droplets may have fallen using appropriate disinfectant products. If the change of work shift also involves the renewal of the staff, it can be considered that sanitization also involves the zeroing of the quanta of infection present in the first shift. If there is no change of personnel, the infected subject may be present also in the second shift. However, it is possible to simulate both cases. It is observed that the continuous trend highlighted for the various quantities is not mandatory at all, as it will be clearer in the next sections considering a variable number of people.

**FIGURE 3:**
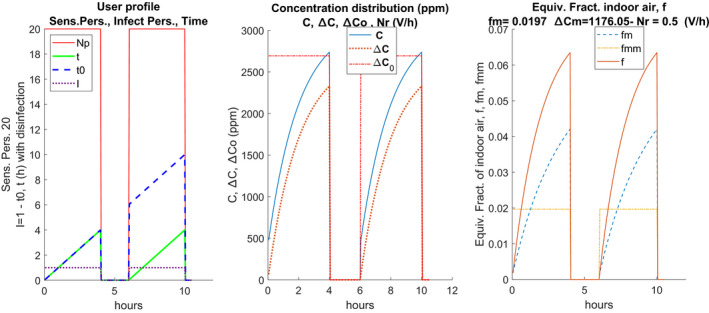
Example of scenario without intermediate disinfection

The central sub‐figure shows the trends of **C**, Δ **C**, and ${\Delta C}_{0}$. The plots are repeated for each air exchange value, $N_{r}$, considered. The right plot shows the hourly trends for each value of $N_{r}$, **f**, $f_{m}$, and $f_{\mathrm{mm}}$, shown in solid and bold lines. In the absence of intermediate sanitization, the values shown in the diagrams of the following Figure 3 are obtained. It can be observed that the CO_2_ concentration always increases even after the pause because the sanitization measures have not been implemented (total air change, cleaning of the surfaces).

Hourly trends are highly dependent on the number of hourly changes, $N_{r}$, as can be observed in the central plot. The hourly trends of the equivalent fraction of indoor air, **f**, and of its average value, $f_{m}$, vary according to the presence or absence of sanitization. The average values are visibly higher than in the current case of sanitization and this also affects the calculation of the probability of individual contagion, as will be seen in the following sections. The term $\Delta =‐C_{0}$ must also be discretized in the form:

(13) $$C‐C_{0}=\frac{C_{G}}{N_{r}V}=\frac{p^{\prime }N_{p}}{V}$$

To calculate the hourly variation of the CO_2_ concentration in the environment, the balance for the CO_2_ concentration can be stated as:

(14) $$V\frac{\mathrm{dC}}{\mathrm{dt}}=Q\left(C_{0}‐C\right)+{\overset{\cdot }{S}}_{g}$$

where dt is the time interval. The transient solution is:

(15) $$C=C_{0}+S_{G}+\left(C_{I}‐C_{0}S_{G}\right)e^{‐N_{r}t}$$

Observe how **t** determines the trend of concentrations with or without sanitization in the event of a double work shift, as illustrated in the previous figures for the scenarios. From the distribution of **C** we obtain ΔC and therefore the discretized values of **f**:

(16) $$f=\frac{\Delta C}{C_{a}}$$

Given the hourly distribution of **f**, its average value is calculated, i.e.

(17) $$f_{m}=\frac{\sum_{0}^{t}f}{\Delta t}$$

used to calculate the probability of contagion. The spatial concentration of the quanta^21^ is given by the expression:

(18) $$N\left(t\right)=\frac{q}{\mathrm{LV}}\left(1‐e^{\mathrm{Lt}}\right)$$

used to calculate the probability of contagion. The spatial concentration of the quanta^21^ is given by the expression:

The average concentration is then obtained from Eq. (18) by integrating and dividing by the time interval Δt:

(19) $$\bar{N}\left(\Delta t\right)=\frac{1}{\Delta t}\int_{0}^{\Delta t}q(t)dt=\frac{q}{\mathrm{LV}}\left[1‐\frac{1}{L\Delta t}\left(1‐e^{‐L\Delta t}\right)\right]$$

The average value $\bar{q}$ of the production of quanta of infection for the whole volume V, calculated in the period of time Δt, is then:

(20) $$\bar{q}\left(\Delta t\right)=q\left[1‐\frac{1}{L\Delta t}\left(1‐e^{‐L\Delta t}\right)\right]$$

where $\bar{q}=\bar{N}LV$.

The mask can also be taken into account using the term $f_{\mathrm{mask}}$, i.e. the reduction factor of the quanta flow due to the mask, as suggested by Gammaitoni and Nucci^16^:

(21) $$f_{\text{mask}}=\left[100‐\frac{\mathrm{XY}}{100}\right]\%$$

The action of the mask is directly applied to the hourly production of quanta coming from the mouth or nose, as indicated in Eq. (21). Then, introducing the reduction factor $f_{\mathrm{mask}}$ in Eq. (20), written in discretized form, yields

(22) $$\bar{q}\left(\Delta t\right)=q\left[1‐\frac{1}{L\Delta t}\left(1‐e^{‐L\Delta t}\right)\right]f_{\text{mask}}$$

where L takes into account the quanta reduction effects due to the use of high‐efficiency filters, UV‐ray lamps, the deposit of quanta on surfaces, and the inactivation of viruses. This factor L is written as^25^, ^26^:

(23) $$L=N_{r}+\lambda_{f}\eta_{f}+\lambda_{\mathrm{UV}}\eta_{\mathrm{UV}}+\lambda_{\mathrm{dep}}+\lambda_{\mathrm{vit}}$$

Fisk and Nazaroff method^25^ assumes that the terms of (23) increase the number of equivalent hourly changes. High‐efficiency filters, where possible, and the use of UV lamps also sanitize the air and affect the model because they vary the hourly distribution of quanta, given in expression (22). Furthermore, the expression for $\lambda_{f}$ ^27^ results in

(24) $$\lambda_{f}\eta_{f}=f_{\mathrm{HVAC}}\frac{Q_{f}\eta_{f}}{V}$$

with $f_{\mathrm{HVAC}}$ denoting the fraction of time in which the HVAC system is in operation. The term $\lambda_{\mathrm{dep}}$ varies from 0.8 to 1.5 (1/h) and can be calculated through the expression^26^, ^28^:

(25) $$\lambda_{\mathrm{dep}}=\frac{0.108d_{p}^{2}\left(1+\frac{0.166}{d_{p}}\right)}{H}$$

**FIGURE 4:**
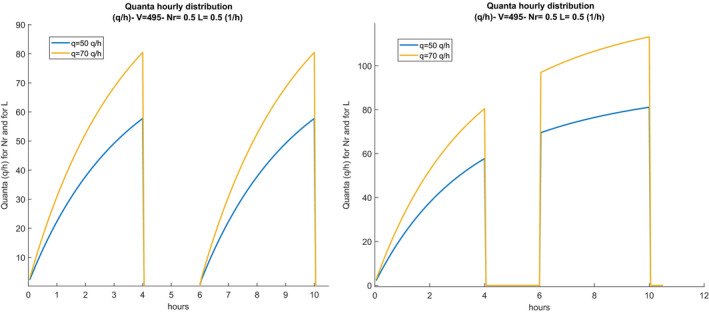
Hourly distribution of quanta production (t) with intermediate sanitization (left) and without sanitization (right)

The value of $\lambda_{\mathrm{vit}}$, also referred to as inactivation, depends on the humidity of the air and varies from 0.5 to 1.2 (1/h). Some researchers ignore this, believing that the epidemiological definition mechanism of quanta automatically takes them into account. The hourly distribution of the quanta also follows the same changes as the distribution of the CO_2_ concentration, as illustrated in Figure 4.

On the right sub‐figure, the hourly distribution of the quanta is not affected by the sanitization and has no interruptions, on the left, there is the intermediate sanitization and the hourly distribution of the quanta restarts from zero in the second work shift.

## 4 INFLUENCE OF THE NUMBER OF CHANGES PER HOUR

**FIGURE 5:**
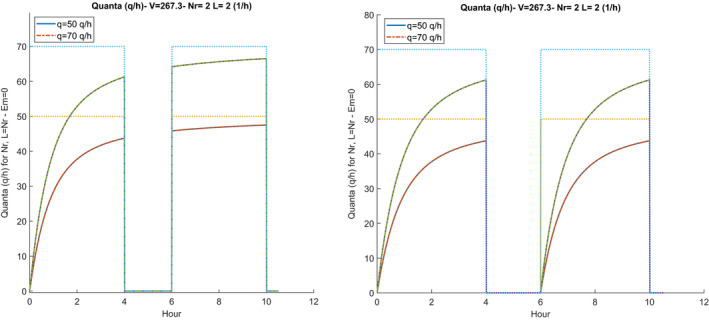
Effect of sanitization on the hourly production of the average quanta of infection: with sanitization (right); without sanitization (left). The dotted lines represent the steady‐state conditions

Here, the distinction between the effective and equivalent number of changes per hour is emphasized. For the calculation of the hourly variations of CO_2_ concentrations, it is always necessary to refer to the effective number of hourly changes, $N_{r}$, since this concentration is used by the Rudnick and Milton method as a marker of respiration and therefore, it would be distorted if it did not refer to the effective air exchange. Contrary to what happens for the Wells‐Riley and Gammaitoni‐Nucci methods, the sanitizing function of the additional devices (high‐efficiency filters, UV lamps, droplet deposit, and reduction of viral vitality) is evaluated by a fictitious increase in the number of virtual air changes.^25^ According to Rudnick and Milton's method, this equivalent number of changes per hour can only be inserted in the expression for calculating the hourly variation of quanta and therefore indirectly inserted in probability, i.e. into Eq. (12). After all, the action of these sanitization mechanisms is precisely to reduce the hourly production of quanta. In Figure 5, the hourly trend of *q* as a result of sanitization (assuming *L* as the modified number of hourly changes), and without sanitization (assuming $N_{r}$ as the effective number of hourly changes), are respectively plotted in solid and dashed lines.

The differences are significant and have a high impact on reducing the risk of airborne infection. Note that the action of the mask acts directly on the hourly production of the quanta emitted from the mouth or nose, see Eq. (11) regardless of the value of *L*.

## 5 BENEFITS OF DYNAMIC CALCULATION WITH THE MODIFIED RUDNICK AND MILLER METHOD. THE DYNAMIC CALCULATION FOR A CLASSROOM

The dynamic calculation brings some benefits in the higher calculation accuracy and adherence to the actual conditions of use and presence in the environment. The hourly distribution of the CO_2_ concentration is the trend reported in Figure S1. The evolution of the transient concentration is faster for high values of the hourly changes and slower for low values of $N_{r}$. In the same figure, it is observed that for $N_{r}$ = 0.5 the values of C stabilize after 9 h while for $N_{r}$ = 3.5 after 2 h.

**FIGURE 6:**
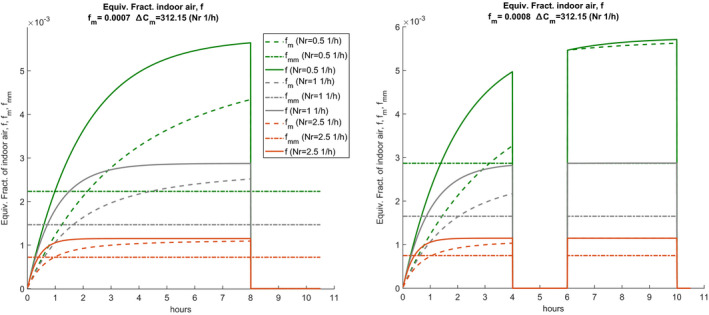
Trend of **f**, of the average variable value $f_{m}$ and total $f_{\mathrm{mm}}$ for single and double shift

Since the factor $f_{m}$ depends on the ratio $\Delta /C_{a}$ also $f_{m}$ will have a transient that follows that of the concentrations, as illustrated previously and again reported in Figure 6, both for single and double work shift with sanitization, where $f_{m}$ has been scaled to fit in the same figure.

It follows that the dynamic calculation carried out using Eq. (12) takes into account the variability of $f_{m}$ in the initial transitory period which will be greater the smaller the number of hourly changes. In the case of double work shifts with intermediate sanitization, the transients restart at the beginning of each shift, and the trend of concentrations and $f_{m}$ are affected considerably. If the variability of the hourly distribution of quanta is also taken into account, see previous figures, we understand how the probability of contagion calculated with the vectorized expression (22) in the dynamic regime is lower than that calculated using Eq. (11) in the constant regime. In general, the steady‐state regime calculates the risk of contagion in excess while the dynamic regime calculates it with greater precision, obtaining more realistic results. A similar observation applies if the Wells‐Riley or Gammaitoni‐Nucci calculation methods are used. Another observation concerns the application of the direct design method in which it is assumed that the difference ΔC is calculated from the ratio between the internal source and the airflow, i.e. considering the steady‐state value of the transients considered above for concentrations. The comparison between variable ΔC in dynamic and steady‐state is given in Figure S2 of the supplementary information.

**FIGURE 7:**
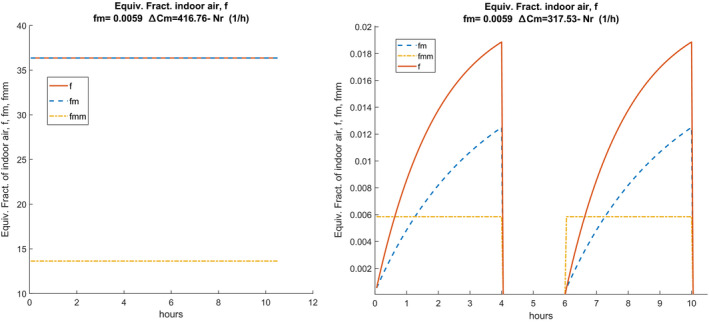
Comparison between the stationary equivalent fraction of indoor air and the dynamic mean value $f_{m}$ for two work shifts with sanitization

The stationary limit value is always significantly higher than the variable one. By imposing a stationary $\Delta C_{0}$, a stationary factor **f** is obtained given by the ratio $\Delta /C_{a}$ while in the dynamic regime **f** varies instantly and the average value ${f_{m}}_{m}$ must be calculated. Figure 7 compares the two cases. Once again, the stationary method overestimates the calculation parameters and therefore the probability of individual contagion risk.

**FIGURE 8:**
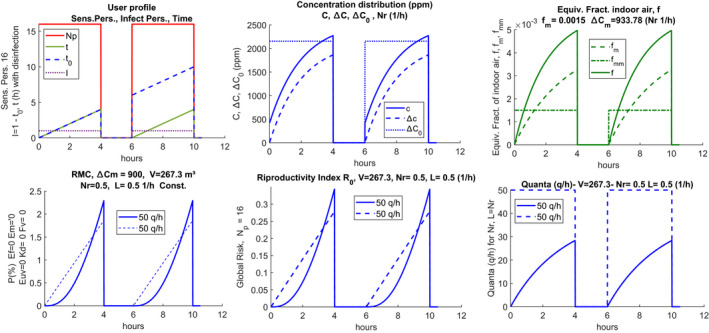
Scenario and risks for high school classroom with double work‐shift and intermediate sanitization, $N_{r}$ = 0.5 (1/h)

**FIGURE 9:**
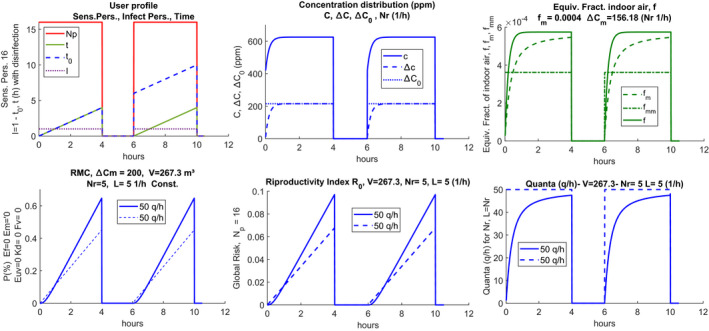
Scenario and risks for high school classroom with double work shift and intermediate sanitization, $N_{r}$ = 5 (1/h)

Figure 8 shows the case of a classroom for high school with the usage profiles^29^ reported in the first row for $N_{r}$ = 0.5 (1/h). The second row reports the probability of individual contagion for a production of 50 (quanta/h) with variable temporal distribution, the reproductivity number, $R_{0}$, also called the probability of global contagion given by the product $(N_{p}‐1)P$, where $N_{p}$ = 17, and finally, the hourly trend of the quanta of infection.^4^ It is observed that there are very high individual contagion probabilities. These are significantly reduced for $N_{r}$ = 5 (1/h), as illustrated in Figure 9. It can be observed, once again, how this hourly distribution reaches the regime condition much faster when the number of hourly changes is higher.

## 6 DYNAMIC ANALYSIS DISCRETIZED WITH A VARIABLE NUMBER OF PEOPLE

The discretized dynamic method allows for considering a variable number of people $N_{p}$ and therefore for assessing the risk of contagion due to the breathing and re‐breathing of the people present in the environment. From a formal point of view, everything remains unchanged in the previously written expressions, just consider that $N_{p}$ is no longer constant and varies over the period considered.

### 6.1 Dynamic calculation for shop

It is considered a shop with an area of 15 × 10 (m^2^), a volume of 495 (m^3^), and 5 employees. Moreover, the air changes per hour are set to $N_{r}=5$. It is assumed, as required by the anti‐COVID 19 regulation in Italy, that the customers present within the shop cannot exceed the internal staff. Three cases are compared in which two opening work shifts of 4 h each are separated by the closure of 2 h and it *t* is assumed that there is sanitization of the air and surfaces during the closing period. The first scenario considers a maximum number of ten people constant for all the simulated time. The second scenario simulates a trend of a statistical type with a variable number of people. Assuming that at the opening only a few customers are present then the number grows more and more until they reach a maximum of ten people at the closing. Of course, this distribution can vary freely. The purpose of examining a case with variable $N_{p}$ is to see if the probability of individual risk decreases for an average presence of less than the expected maximum number of ten people. Finally, the third scenario is similar to the second one but it takes into account only the air sanitization during the pause between work shifts but not the sanitization of quanta/h, i.e. it is assumed that the infected can remain even in the second shift and therefore the hourly production of quanta remains unchanged.

**FIGURE 10:**
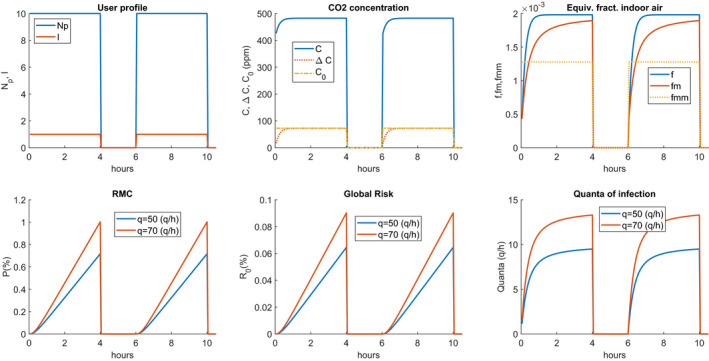
Store example case: first scenario with constant $N_{p}$ and total sanitization

**FIGURE 11:**
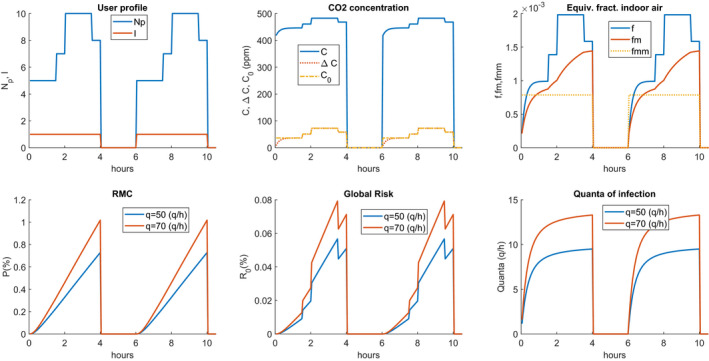
Store example case: second scenario with variable $N_{p}$ and total sanitization

Comparing the two scenarios reported in Figures 10 and 11, it is observed that the variation in the number of people, $N_{p}$, is immediately reflected on the concentration of exhaled CO_2_ and the calculated difference Δ. The $f_{m}$ also varies accordingly. The comparison between the two cases with constant and variable $N_{p}$ shows how the number of people modifies the probability of global contagion $R_{0}$ but not the probability of contagion P which is independent of $N_{p}$ as remarked in Section 3.

### 6.2 Dynamic calculation for office

**FIGURE 12:**
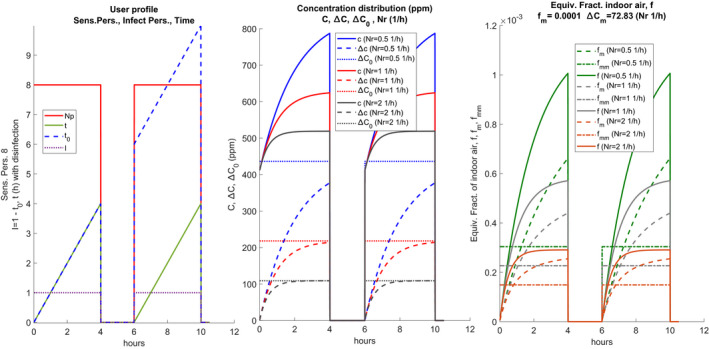
Scenario of an office with 8 people

**FIGURE 13:**
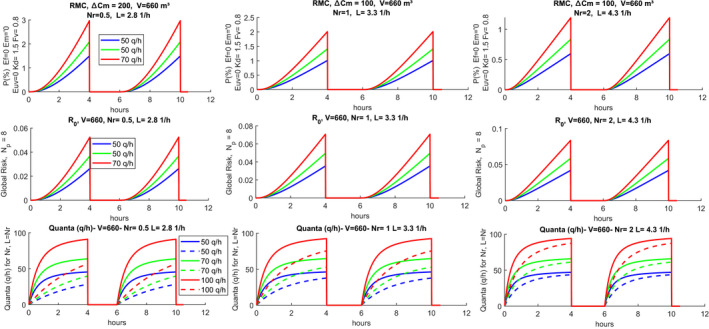
Probability of individual and global contagion with air sanitization only and for variable $N_{r}$

We want to examine the case of an office of 10 × 20 (m^2^), with 8 internal employees always present in two shifts of 4 h each and a break of 1 h. The sanitization of the air but not of the quanta is provided, that is, it is assumed that the infected person may be present both in the first and second shift. A modified version of the calculation program is used which simultaneously analyzes three air exchange conditions, 0.5, 1, and 2 (1/h), and three hourly productions of quanta: 40, 70, and 100 (q/h). The user profile is shown in Figure 12 with the usual information on the profile of people, infected people, concentrations, and the fraction of breathing air. Figure 13 shows the probabilities of individual contagion in the first row for the three values of $N_{r}$, of the global probability in the second row as the number varies and in the third row there is the hourly distribution of quanta. The use of protective masks with frontal efficiency of 90% was assumed and that the deposit of droplets on the floor and the decrease in virus vitality are considered. The hourly distribution curves of the quanta show both the curves for real $N_{r}$ (dashed) and for corrected $N_{r}$ (with the value of ***L***, in solid line). The comparison for the three cases of $N_{r}$ leads to consider the probabilities of contagion as acceptable for $N_{r}$ = 2 (1/h).

## 7 INVERSE PROBLEM: DETERMINATION OF THE QUANTA OF INFECTION. SUPER‐SPREADING EVENT IN THE SKAGIT VALLEY CHORALE

The possibility of multiple comparisons with multiple values of the hourly production of quanta, **q**, and for multiple values of air changes, $N_{r}$, is useful for the reverse work of identifying the quanta of infections produced. In this hypothesis, having known the number of infected in an environment, it is possible to estimate, by multiple attempts, the value of the quanta that have been produced for the infection. This identification has the advantage of being carried out using the dynamic analysis and therefore with the most correct and realistic calculation conditions compared to any calculation method with constant parameter values. In the presence of a CO_2_ sensor, the abacuses of the distributions of **C**, ΔC, and $\Delta C_{0}$ reported in the usage profiles can be employed to identify the real internal conditions of the environments and then evaluate the consequent risk probabilities. For the sake of simplicity, surface contact propagation and the influences of temperature and humidity are not considered in this case.

Miller et al.^21^ describe a case of superspreading in Skagit Valley Chorale, Washington state, which occurred on March 10, 2020. On that occasion, 53 out of 61 singers were infected with COVID‐19. The authors, based on the findings made and the facts ascertained, summarize the calculation parameters in the following Table 1.

**TABLE 1:** Calculation values for the simulations of the Skagit Valley Chorale^21^

Parameter	Value(s)	Distribution
Probability of infection, *p* (%)	53–87	Uniform
Volumetric breathing rate, $Q_{b}$ (m^3^/h)	0.65–1.38	Uniform
Loss rate due to ventilation, $\lambda_{v}$ (h^−1^)	0.3–1.0	Uniform
Loss rate due to deposition onto surfaces, $\lambda_{\mathrm{dep}}$ (h^−1^)	0.3–1.5	Uniform
Loss rate due to virus inactivation, *k* (h^−1^)	0–0.63	Uniform
Volume of rehearsal hall, *V* (m^3^)	810	Constant
Duration of rehearsal, Δt (h)	2.5	Constant
Volumetric inhalation rates of singers, *p*, (m^3^/h)	0.22–1.38	Uniform

This is an anomalous case that occurred during some choral rehearsal sessions inside a building of 180 × 4.5 = 810 (m^3^) in which, among the 61 present (60 of the choir plus one employee), there were 53 cases of suspected contamination with 33 confirmed cases. The building appeared poorly served from a plant engineering point of view. There was a 20° (C) hot air generator whose operation it is unknown whether it was continuous or discontinuous. In any case, it was clear to the researchers who studied the case that the air ventilation was lacking, see Table 1. Many calculation parameters are imprecise and cannot be correctly determined afterward. Moreover, the bibliography indicated for each of them is rather vague and imprecise. Therefore, the simulations that can be done must take into account a significant variability of some fundamental parameters, as reported in Table 1. The Authors used the Gammaitoni‐Nucci expression^16^ for the calculation of the spatial concentration of quanta, (quanta/m^3^), and the hourly production, (quanta/h), as described in Section 3.2. Here, we want to perform an inverse calculation for the determination of the hourly production of quanta using the data available in the original publication.^21^ In the first calculation phase, the CO_2_ concentration produced by the 61 present in the theater hall is determined to determine the difference ΔC required by the calculation method. We do not know the ventilation airflow or the layout of the HVAC system used. It seems that there is a heating system with a heat generator that sends hot air into the theater to maintain a nominal temperature of 20°, although some of those present said they felt cold. The operation of the system, therefore, appears without a thermostat. We have no information on the external airflow or whether there is internal recirculation. The estimated number of air changes varies from $N_{r}$ = 0.3 to $N_{r}$ = 1 (1/h), see Table 1. The same researchers considered these values low, urging the need for effective controlled mechanical ventilation. Furthermore, the ventilation flow rate corresponding to the indicated range also entails a lack of control on ΔC which, as will be seen in the following calculations, will always be high. The researchers reported the lack of a CO_2_ detector for air quality control. Choral activity is equivalent to speaking aloud and therefore droplet emission is quite high. In the aftermath, using data available in the literature, the researchers suggest a volumetric respiration rate ranging between 0.65 and 1.38 (m^3^/h). For comparison, it should be borne in mind that breathing in normal conditions (light office work activity) corresponds to a flow rate of 0.48 (m^2^/h). The volumetric composition of inhaled and exhaled air is shown in Table 2:

**TABLE 2:** Air composition

Inspired air	Composition/Gas	Expired air
21%	Oxigen	17%
78%	Nitrogen	78%
~0%	Carbon dioxide	4%
1%	Various gases traces	1%

Therefore, the exhaled CO_2_ flow rate will be equal to 4% of the breathed airflow. Based on the activity carried out by the singers and the high breathing rate, a CO_2_ production of 0.4 (L/h) was considered. The value of the re‐breathing fraction is equal to $C_{a}$ = 47,500 (ppm). The verification criteria already presented previously will be followed to identify the hourly production of quanta. It must be said that the variability of some parameters does not allow the direct and univocal calculation of *q* but it is necessary to carry out several iterations. With the verification criterion the presence of any filters is not taken into account, the difference $\Delta C=C‐C_{0}$ is calculated as a function of the ventilation flow‐rate (in the range indicated in Table 1) by means of the Eq. (15) from which the calculation **f** follows by Eq. (16). Calculated the average value of **f**, the vectorized dynamic Rudnick and Milton Eq. (12) is used, modified by Fisk and Nazaroff according to Eq. (23), even if there are no protective masks or filtration of the recirculation air with HEPA filters. From the abacuses obtained, for the exposure time $T_{l}$ = 2.5 (h), assuming that the selected parameters are kept constant and that steady‐state conditions stand, the range of variation of *q* for each hypothesis can be obtained. The real solution cannot be univocal due to the uncertainties indicated in Table 1. The variability of *q* appears considerable, as reported by the researchers in their publication. The parameters adopted for the calculation have averaged values in the intervals indicated in the article^21^ and are reported in Table 3:

**TABLE 3:** Parameters used to simulate the Skagit Valley Chorale scenario

Parameter	Value(s)
Volumetric breathing rate, $Q_{b}$ (m^3^/h)	1.1
Volumetric CO_2_ expiration, $Q_{b}$ (m^3^/h)	0.28
Loss rate due to ventilation, $\lambda_{v}$ (h^−1^)	0.3, 0.6, 1.0
Loss rate due to deposition onto surfaces, $\lambda_{\mathrm{dep}}$ (h^−1^)	0.5
Loss rate due to virus inactivation, *k* (h^−1^)	0.3
Volume of rehearsal hall, *V* (m^3^)	810
Number of singers inside, $N_{p}$ (−)	61
Quanta of infection, (q/h)	600, 1200, 1800, 2400

**FIGURE 14:**
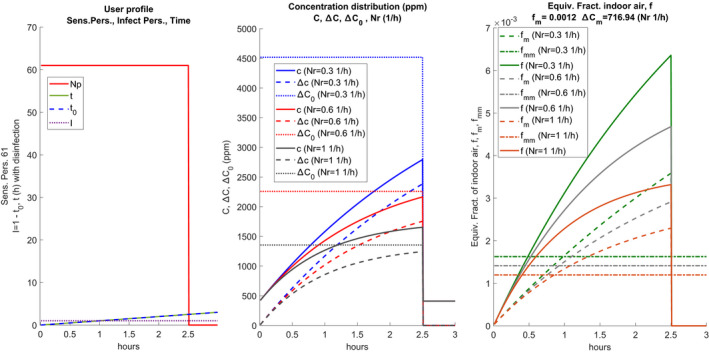
Simulation scenario for the Skagit Valley Chorale

**FIGURE 15:**
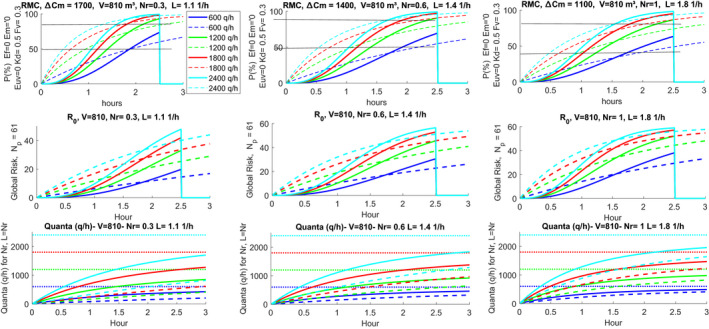
Calculation of individual and global probabilities and hourly distribution of quanta. The static cases are plotted in dashed‐lines

The results for the scenario are shown in Figures 14 and 15 where a 2.5‐h guideline is also shown. It is observed that the value of $\Delta C_{0}$ is 3000 (ppm) for 1.0 (V/h), 5000 (ppm) for 0.6 (1/h) and 10,000 (ppm) for 0.3 (1/h) for hourly changes. These are high values, well above 800 (ppm) indicated by ASHRAE standards as the limit value for air quality. This means that the mechanical ventilation was either off or completely inadequate for air quality control. The values of Δ vary considerably during the singing trial period. The calculations of the probability of individual and global contagion and the hourly distribution of quanta are reported in Figure 15. The latter contains the guidelines for the time of 2.5 h and the individual contagion probability values of 53% and 87% corresponding to ratios 32/60 and 52/60, considering one initial infected.

From the examination of the first abacuses of each row, respectively, for 0.3, 0.6, and 1 (1/h), in correspondence with time *t* = 2.5 (hours) and at the probability lines of 53% and 87%, we have the values of the corresponding hourly quanta. By interpolating between the curves, the following summary of the (quanta/h) corresponding to the infected indicated is obtained.

If we observe the calculation results with the static and dynamic results both present we can make some observations. Static simulations overestimate the calculation results and therefore provide risk probabilities with lower quanta/h. If we compare the results with the dynamic method and the analogs with the static method, we have the following Table 4.

**TABLE 4:** Variability of quanta/h for the dynamic and static calculations

Probability (%)	Infected (N)	Dynamic calculation			Static calculation		
		q/h (ACH 1/h)			q/h (ACH 1/h)		
		0.3	0.6	1.0	0.3	0.6	1.0
87	53	950	1000	1200	1350	1600	1800
53	33	350	390	450	500	600	700

Summarizing, Table 5 reports the variability of quanta/h, expressed in terms of minimum, maximum, and mean value, for the two types of calculations of Table 4.

**TABLE 5:** Summary of the quanta/h produced in the dynamic and static cases

Probability (%)	Infected (N)	Dynamic calculation			Static calculation		
		q/h (ACH 1/h)			q/h (ACH 1/h)		
		Min	Max	Mean	Min	Max	Mean
87	53	950	1200	1075	1350	1800	1575
53	33	350	450	400	500	700	600

The average value of quanta production for the dynamic calculation is 738 (q/h) while for the static calculation it is 1088 (q/h). The results obtained in Ref. [21] are compared with the values calculated using the proposed method, as indicated in the Table 6;

**TABLE 6:** Comparison of results of the proposed method with those reported in Ref. [21].

Data	Dynamic calculation			Static calculation		
	q/h (ACH 1/h)			q/h (ACH 1/h)		
	Min	Max	Mean	Min	Max	Mean
Miller et al.^21^	550	1510	970	550	1510	970
Proposed method	400	1075	738	600	1575	1088

There is a substantial agreement for the minimum values of the quanta/h (especially for the static method) while there is a difference of 12.2% for the maximum values. The differences are certainly due to the calculation method used in the two cases: dose‐reaction with 1000 Monte Carlo simulations to solve the dispersion of the input data for Miller's article versus a single simulation for the dynamic method proposed here. However, it must be considered that, given the great variability of the input data, both methods show considerable uncertainty and dispersion in the calculation of the quanta rate.

## 8 CONCLUSIONS

The proposed discretized dynamic method offers considerable advantages over the traditional methods in use. These can be summarized as follows:

1. Possibility of using scenarios to better characterize the presence of people inside the rooms, the use of the rooms (double work shifts with intermediate break), and the possibility of sanitizing the rooms (total air exchange, air sanitization using UV‐lamps, disinfection surfaces). The recent paper^30^ uses only part of the proposals reported in this paper that have found application in the previous paper^29^;
2. Possibility of enabling or not the sanitization of quanta in the case of double work shifts with the same staff in the presence of probable infection.
3. Ability to perform calculations using progressive average values instead of global average values. This allows for more accurate and reliable results. Methods that use constant values overestimate the final results when calculating the probability of contagion.
4. The proposed method etiologically correlates the cause (re‐breathing) to the effect (contagion) while the traditional methods are affected only by external conditions and do not take into account the presence of people inside.
5. The calculation of the CO_2_ concentrations can be used, indirectly, to verify, following **EN 16788‐1**, the indoor air quality (IAQ) in the rooms.

The case studies discussed in the paper have demonstrated that carrying out an intermediate environmental sanitization to remove the initially infected sources and eliminate the CO_2_ production of the previous work shift significantly reduces the risk of individual contagion.

## Nomenclature

*C*
: CO_2_ concentration in the indoor air, (ppm)
*C*_0_
: CO_2_ concentration in the outside air, (ppm)
*C_a_*
: CO_2_ concentration in the air exhaled during breathing, evaluated between 37000 and 45000, (ppm)
*C_I_*
: initial concentration of CO_2_ in the indoor air, (ppm)
*dp*
: aerodynamic diameter of the droplets, (mm)
f, fm, fmm: respectively, the equivalent fraction of indoor air, the average value, and the global average value over the calculation time
fHVAC: fraction of time in which the HVAC system is in operation
fmask: reduction factor of the quanta flow due to the mask
H: height of the room, (m)
I: the number of initial infected
*L*
: air changes per hour taking into account the quantum reduction effects, (1/h)
N: total concentration of the quanta of infection, (quanta/m^3^)
${\bar{N}}_{p}$
: average number of people inside the volume
*N_p_*
: number of people inside the volume
*N_r_*
: air changes per hour or ACH, (1/h)
*p*
: breathing activity of ambient air of a single individual, (m^3^/h)
P: probability of contagion
p′: flow rate of CO_2_ emitted for ventilation, (m^3^/h)
$\bar{q}$
: average emission rate or average value of the production of quanta of infection, (quanta/h)
*q*
: nominal emission rate or nominal value of the production of quanta of infection within the environment, (quanta/h)
Q: the total flow rate of fresh air, (m^3^/h)
*R*_0_
: probability of global contagion
*S_g_*
: internal source of CO_2_, (mL/h)
*t*, *t*_0_
: respectively the exposure time from entering the environment and the progressive time, (h)
V: volume of the environment, (m^3^)
*V_e_*
: equivalent volume of exhaled air contained in the environment, (m^3^)
X, Y: frontal and lateral mask efficiencies
Δ*C*, Δ*C*_0_
: respectively the CO_2_ concentration difference between inside and outside in dynamic and steady‐state conditions, (ppm)
*n_f_*
: efficiency of the filtration varying from 0.9 to 0.99
η_UV_
: UV‐rays efficiency varying from 0.8 to 0.99
*λ*_dep_
: equivalent number of ACH for the deposit of droplets
*λ_f_*
: equivalent number of additional hourly changes per filtration
*λ*_UV_
: equivalent number of ACH due to UV‐rays
*λ*_vit_
: equivalent number of ACH for reduction of viral vitality

### PEER REVIEW

The peer review history for this article is available at https://publons.com/publon/10.1111/ina.12862.

## Supporting information

Figure S1

Figure S2

## Data Availability

The data that support the findings of this study are available from the corresponding author upon reasonable request.
